# Integrated clinico-molecular profiling of appendiceal adenocarcinoma reveals a unique grade-driven entity distinct from colorectal cancer

**DOI:** 10.1038/s41416-020-1015-3

**Published:** 2020-07-31

**Authors:** Kanwal Raghav, John P. Shen, Alexandre A. Jácome, Jennifer L. Guerra, Christopher P. Scally, Melissa W. Taggart, Wai C. Foo, Aurelio Matamoros, Kenna R. Shaw, Keith Fournier, Michael J. Overman, Cathy Eng

**Affiliations:** 1 Department of Gastrointestinal Medical Oncology, The University of Texas MD Anderson Cancer Center, Houston, TX USA; 2 Department of Surgical Oncology, The University of Texas MD Anderson Cancer Center, Houston, TX USA; 3 Department of Pathology, The University of Texas MD Anderson Cancer Center, Houston, TX USA; 4 Department of Diagnostic Radiology, The University of Texas MD Anderson Cancer Center, Houston, TX USA; 5 Institute for Personalized Cancer Therapy, The University of Texas MD Anderson Cancer Center, Houston, TX USA

**Keywords:** Tumour biomarkers, Gastrointestinal cancer

## Abstract

**Background:**

Appendiceal adenocarcinoma (AA) is an orphan disease with unique clinical attributes but often treated as colorectal cancer (CRC). Understanding key molecular differences between AA and CRC is critical.

**Methods:**

We performed retrospective analyses of AA patients (*N* = 266) with tumour and/or blood next-generation sequencing (NGS) (2013–2018) with in-depth clinicopathological annotation. Overall survival (OS) was examined. For comparison, CRC cohorts annotated for sidedness, consensus molecular subtypes (CMS) and mutations (*N* = 3283) were used.

**Results:**

Blood-NGS identified less *RAS/GNAS* mutations compared to tissue-NGS (4.2% vs. 60.9%, *P* < 0.0001) and showed poor concordance with tissue for well-/moderately differentiated tumours. *RAS* (56.2%), *GNAS* (28.1%) and *TP53* (26.9%) were most frequent mutations. Well/moderately differentiated tumours harboured more *RAS* (69.2%/64.0% vs. 40.5%) and *GNAS* (48.7%/32.0% vs. 10.1%) while moderate/poorly differentiated tumours had more *TP53* (26.0%/27.8% vs. 7.7%) mutations. Appendiceal adenocarcinoma (compared to CRC) harboured significantly fewer *APC* (9.1% vs. 55.4%) and *TP53* (26.9% vs. 67.5%) and more *GNAS* mutations (28.1% vs. 2.0%) (*P* < 0.0001). Appendiceal adenocarcinoma mutation profile did not resemble either right-sided CRC or any of the four CMS in CRC. High grade, but no mutation, was independently predictive of survival.

**Conclusion:**

Integrated clinico-molecular profiling of AA identified key molecular drivers distinct from CRC. Appendiceal adenocarcinoma has a predominantly grade-driven biology that trumps mutations.

## Background

Appendiceal adenocarcinoma (AA) is an orphan malignancy (estimated 1 new case per 100,000 person per year) with unique clinical attributes that are distinct from colorectal cancer (CRC) (see Data Supplement Table S1).^1,2^ Appendiceal adenocarcinomas are characterised by a clinical spectrum that ranges from indolent to aggressive behaviour driven predominantly by pathological features.^3,4^ Appendiceal adenocarcinomas have traditionally been described under one of three prognostic histological subtypes: mucinous, non-mucinous and signet-ring cell with better survival observed in the mucinous and non-mucinous subtypes (5-year overall survival (OS): 61% and 53%, respectively) compared to signet-ring cell carcinomas (28%).^1^ The interplay of grade (well, moderate and poor) with TNM stage has emerged as a governing prognostic-predictive factor and has been uniquely incorporated in the AJCC/TNM staging system.^3,4^

Despite distinctive features, the rarity of AAs has limited dedicated research efforts. Due to lack of both clinical and pre-clinical data specifically for AAs, current management guidelines for AAs are often derived from evidence used to manage CRC. This is based on an assumption of biological similarity due to anatomic vicinity and common embryological origin (i.e. the caecal diverticulum).^5,6^ Nevertheless, the dissimilar epidemiological and clinical behaviour of AA and CRC has led to the proposition that AAs and CRCs are distinct molecular entities and recent studies have supported this reasoning.^7,8^ However, prior molecular analyses have been limited by a lack of clinical and histological annotation and small sample sizes, thereby confining definitive conclusions about the clinico-molecular identity of this disease.^7–21^ Moreover, given the heterogeneity of CRC with regards to sidedness and recently recognised consensus molecular subtypes (CMS), establishing molecular parallels between AA, CRC sidedness and CRC CMS is vital.^22,23^

In this study we hypothesised that AA has a distinct molecular profile compared to both CRC as a whole or any of CMS and that this clinico-molecular profile may impact survival. To test these hypotheses, we performed molecular characterisation (using next-generation sequencing (NGS)) of a large AA cohort with robust clinicopathological annotation and compared it with similar data available for CRC.

## Methods

### Patients and tumour samples

**Fig. 1:**
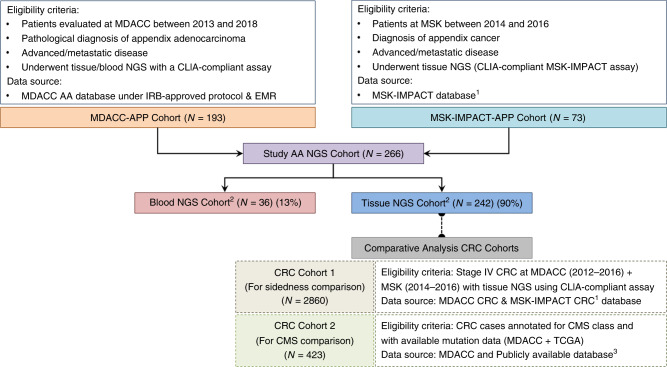
Study Cohorts. Flow diagram illustrating patient selection. AA appendiceal adenocarcinoma, APP appendix, CLIA clinical laboratory improvement amendments, CMS consensus molecular subtype, CRC colorectal cancer, EMR electronic medical records, IRB institutional review board, MDACC MD Anderson Cancer Center, Houston, TX, USA, MSK Memorial Sloan Kettering, New York, NY, USA, NGS next-generation sequencing, TCGA The Cancer Genome Atlas. Note: 1. https://www.cbioportal.org/study/summary?id=msk_impact_2017. 2. Twelve patients had both tissue and blood testing. 3. https://www.cancer.gov/about-nci/organization/ccg/research/structural-genomics/tcga.

Cohort selection for the study is summarised in Fig. 1. The AA cohort consisted of patients diagnosed with metastatic AA (*N* = 193) evaluated at MD Anderson Cancer Center (MDACC) between 2013 and 2018 who were enrolled onto an institutional review board-approved prospective protocol for genomic profiling. Eligible patient had either tumour tissue or blood NGS (or both) performed successfully using a CLIA-compliant assay ordered at the discretion of their treating physician. To avoid confounding comparisons and prevalence across grade that can be affected by tumour cellularity, only patients with a complete NGS test were included (patients with insufficient tissue for NGS and indeterminate NGS results were excluded). This AA cohort was supplemented by additional AA patients (*N* = 73) from a publicly available Memorial Sloan Kettering Cancer Center MSK-IMPACT database (2014–2016) with available tissue NGS using CLIA-compliant MSK-IMPACT assay.^24^ For CRC sidedness comparison, we used a cohort of metastatic CRC annotated for sidedness from MDACC (2012–2016) and MSK (2014–2016) with tissue NGS using CLIA-compliant assay (*N* = 2860). For CRC CMS comparison, we used a cohort of metastatic CRC annotated for CMS with available mutation data at MDACC and TCGA (The Cancer Genome Atlas) (*N* = 423). Clinicopathological data were retrieved using publicly available database or electronic medical records.

### Molecular analyses

Tissue NGS was performed using multiple CLIA-compliant platforms: Ion Ampliseq Cancer Panel (Life Technologies, Grand Island, NY) (CMS 46/50), Oncomine Comprehensive Cancer Panel assay (ThermoFisher, Waltham, MA), Ion AmpliSeq Comprehensive Cancer Panels (ThermoFisher) (CMS400) performed at MDACC Molecular Diagnostic Laboratory, FoundationOne, MSK-IMPACT.^24–28^ Blood NGS was performed using Guardant360 (Guardant Health, Redwood City, CA) circulating cell-free DNA (cfDNA) testing.^29^ For the comparative exploratory analyses with CRC, sidedness and CMS profiles were extracted using CRC patients reported previously by MDACC, MSK and includes those from TCGA.^22–24^ The genetic profile was extracted using disclosed data from these CLIA tests for the patients.

### Statistical analysis

Descriptive statistics were used to summarise the clinicopathological data. Prevalence of GAs was calculated using mutations reported in the clinical tests for the patients who had successfully completed the NGS testing. The categorical variables were compared by the chi-squared (*χ*^2^) test and Fisher’s exact test. Overall survival was defined as the time elapsed from the date of diagnosis to the date of death from any cause. Survival was estimated by the Kaplan–Meier method and compared by log-rank test. Prognostic factors were assessed by multivariate analysis using the Cox proportional hazards model, and two-sided *P* values (uncorrected for multiple testing) of <0.05 were considered to indicate statistical significance. The analyses were performed with SPSS 24.0 software and GraphPad Prism 8.0. Patients at MDACC consented to participate in this study as approved by the institutional review board.

## Results

### Baseline clinicopathological characteristics

Between 2013 and 2018, a total of 266 patients underwent NGS (Fig. 1). In the AA NGS cohort (see Data Supplement Table S2), median age was 53 years and 54.0% patients were women. Pathologic grading was reported in 192 (72.2%) cases: well-differentiated 54 (28.1%), moderately differentiated 55 (28.7%) and poorly differentiated 83 (43.2%). Cytoreductive surgery (CRS) with heated intraperitoneal chemotherapy (HIPEC) was performed in 58% of patients (median peritoneal carcinomatosis index (PCI) of 17 and 79% patients had completion of cytoreduction score (CCS) 0/1) and 74% patients received systemic chemotherapy (see Data Supplement Table S2).

### Comparison of blood vs. tissue sequencing

**Fig. 2:**
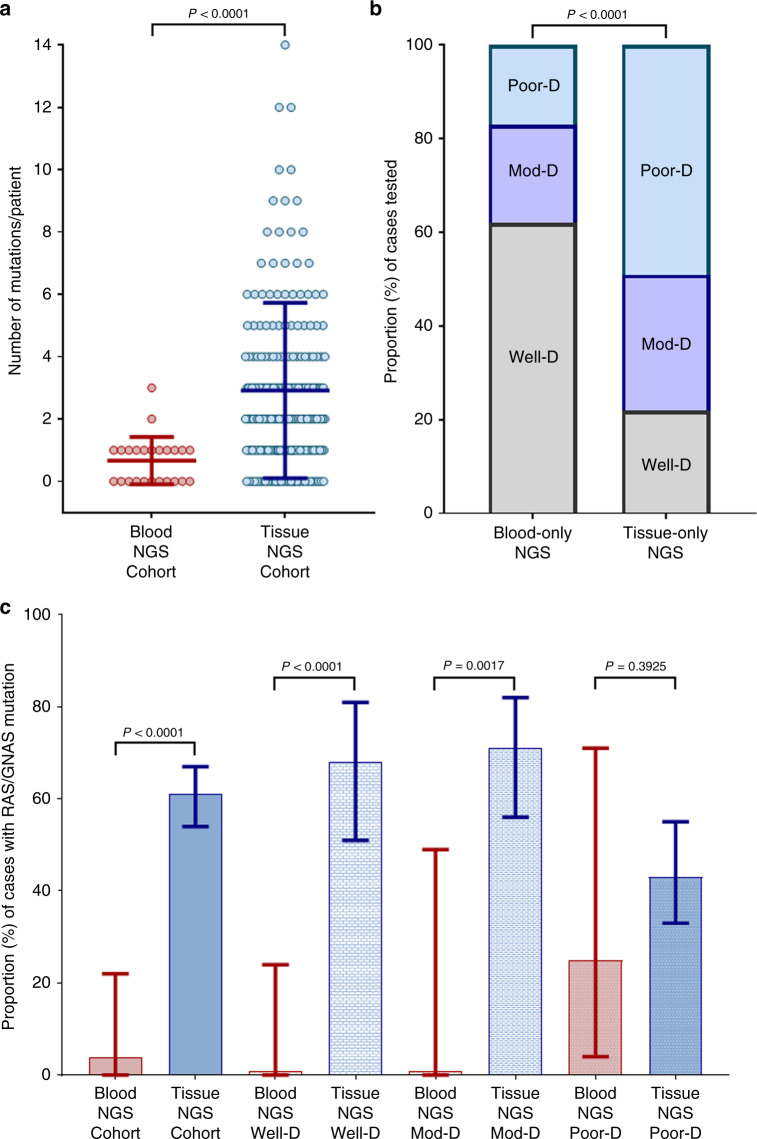
Tissue and Blood Sequencing in Appendiceal Adenocarcinoma. Comparison between blood and tissue NGS vis-à-vis number of mutations per patient (**a**) and proportion of cases with *RAS/GNAS* mutations stratified by grade (**c**). (**b**) shows impact of grade in selection of patients for blood-only and tissue-only NGS. D differentiated, Mod moderately, NGS next-generation sequencing, Poor poorly.

Of the 266 patients in AA NGS cohort, 230 (86.5%), 24 (9.0%) and 12 (4.5%) patients underwent NGS for tissue only, blood only and both, respectively. Between 24 and 230 patients that underwent blood and tissue NGS alone, median number of mutations per patient was significantly lower with blood compared to tissue NGS (1 vs. 2, *P* < 0.0001) (Fig. 2). Patients with well-differentiated tumours were more likely to get blood-only NGS over tissue-only NGS (62.5% vs. 21.8%; OR 5.98, 95%CI: 2.4–13.9, *P* < 0.0001) compared to those with moderate/poorly differentiated tumours (Fig. 2). No specific reason for the choice of test such as tissue availability or quantity/cellularity was discernible. The proportion of patients with *RAS/GNAS* mutations identified by blood NGS (4.2%, 95%CI: 0.0–21.9) was also significantly lower compared to tissue NGS (60.9%, 95%CI: 54.4–66.9) (OR 0.03, 95%CI: 0.0–0.2, *P* < 0.0001) (Fig. 2). This discrepancy between proportion of patient with *RAS/GNAS* mutations identified by blood compared to tissue NGS was seen more in well-differentiated (0.0% vs. 67.7%; OR 0.00, 95%CI: 0.0–0.4, *P* < 0.0001) and moderately differentiated (0.0% vs. 71.1%; OR 0.00, 95%CI: 0.0–0.4, *P* = 0.0017) tumours as compared to poorly differentiated tumours (25.0% vs. 43.4%; OR 0.33, 95%CI: 0.0–2.2, *P* = 0.393).

Among 12 patients that underwent both blood and tissue NGS, concordance analysis using mutations detected at least once in either tissue or blood, revealed a kappa of 0, indicating poor agreement between blood and tissue NGS results. Agreement was seen in only 2/12 (16.7%) cases with regards to absence of mutations. Remarkably, blood testing failed to identify mutations seen on tissue NGS in all remaining ten cases (seven of these ten (70.0%) cases were patients with low-grade AAs). Due to this seemingly low sensitivity of blood NGS for detecting mutations in AA, further analyses were restricted to tissue NGS.

### Mutational landscape of AA

**Fig. 3:**
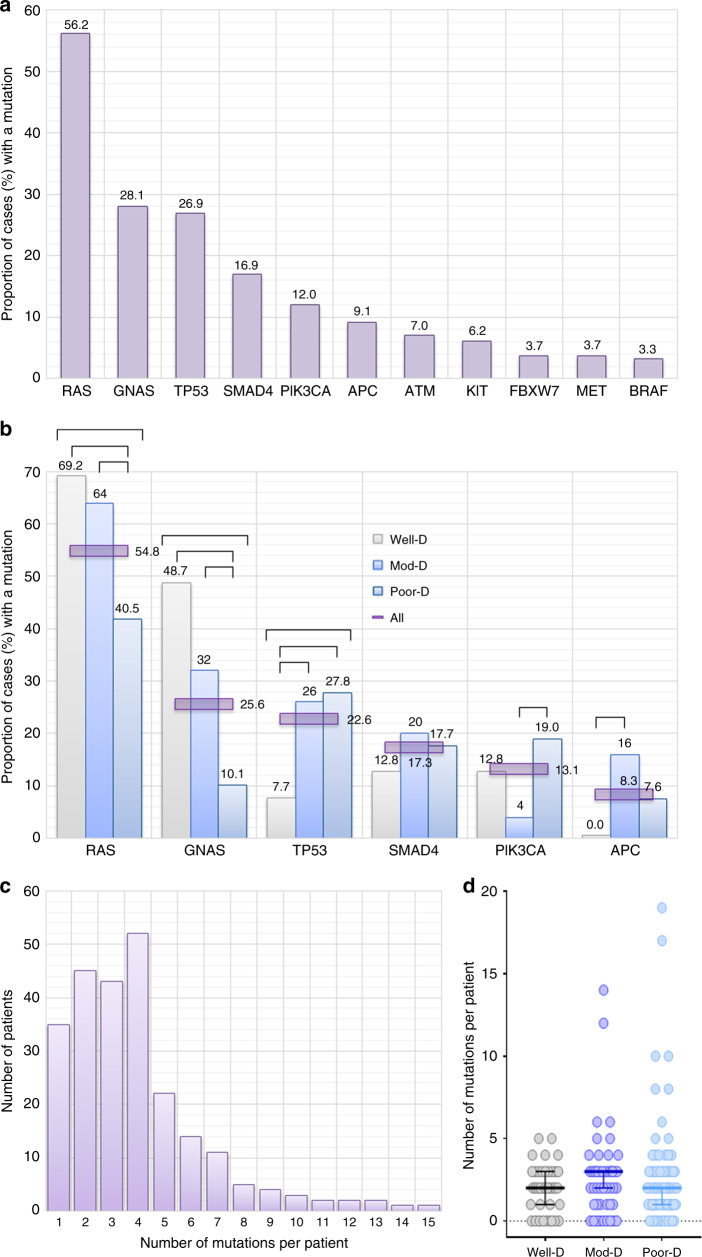
Mutational Profile of Appendiceal Adenocarcinoma. Frequency (%) of mutations (>3% incidence) in tumour tissue from patients with appendix cancer (**a**), distribution of mutation (top six most frequently mutated genes) by grade/differentiation (**b**), distribution of number of mutations per patient (**c**) and distribution of number of mutations per patient by grade/differentiation (**d**). Comparisons are shown for only significant differences (*P* < 0.05). D differentiated, Mod moderately, Poor poorly.

Among 242 patients, 105 distinct genes were found altered (see Data Supplement Table S3). At least one mutation was found in 207 patients (85.5%, 95%CI: 80.5–89.5) with a median of two mutations per patient (range 0–19) and did not vary with the grade of tumour (Fig. 3). The most frequently mutated genes in MDA, MSK and entire cohort were *RAS* (*KRAS* or *NRAS*) (56.2%, 95%CI: 49.9–62.3), *GNAS* (28.1%, 95%CI: 22.8–34.1), *TP53* (26.9%, 95%CI: 21.7–32.8), *SMAD4* (16.9%, 95%CI: 12.7–22.2), *PIK3CA* (12.0%, 95%CI: 8.4–16.7) and *APC* (9.1%, 95%CI: 6.0–13.4) (Fig. 3) (see Data Supplement Table S3 and Supplementary Fig. S1). Prevalence of these mutations varied with grade of tumours. Well- and moderately differentiated tumours were enriched for *RAS* (OR 3.31, 95%CI: 1.4–7.2, *P* = 0.0058 and OR 2.61, 95%CI: 1.2–5.4, *P* = 0.0116) (*P*_trend_ = 0.0033) and *GNAS* (OR 7.67, 95%CI: 2.9–20.0, *P* < 0.0001 and OR 4.18, 95%CI: 1.6–10.6, *P* = 0.0026) (*P*_trend_ < 0.0001) compared to poorly differentiated tumours (Fig. 3) (see Data Supplement Table S4). In contrast, well-differentiated tumours had lower rate of *TP53* mutations compared to moderate- and poorly differentiated tumours (OR 0.24, 95%CI: 0.1–0.9, *P* = 0.0290 and OR 0.22, 95%CI: 0.1–0.7, *P* = 0.0154) (*P*_trend_ < 0.0383) (Fig. 3) (see Data Supplement Table S4). Microsatellite status was assessed in 115 patients; only one patient (0.9%, 95%CI: 0.0–5.2) was found to have microsatellite instability.

### Survival analysis

**Fig. 4:**
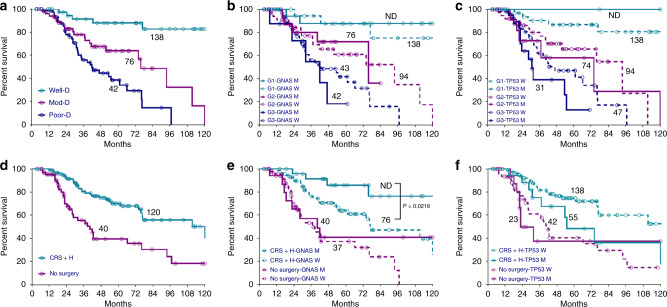
Survival Analyses of Key Prognostic Factors. Kaplan−Meier overall survival (OS) curves for all patients by grade (**a**), and comparison between grade and *GNAS* status (**b**), grade and *TP53* status (**c**), cytoreductive surgery and HIPEC (**d**), cytoreductive surgery and HIPEC and *GNAS* status (**e**), cytoreductive surgery and HIPEC and *TP53* status (**f**). Only statistically significant comparisons are shown. CRS + H cytoreductive surgery and HIPEC, D differentiated, Mod moderately, MUT mutant, ND not defined, Poor poorly, WT wild type.

With a median follow-up of 44.6 months, the median OS for the entire cohort was 75.8 months (95%CI: 58.1–93.5) (see Data Supplement Fig. S2). In univariate analysis, higher tumour grade, lack of CRS with HIPEC, *GNAS* wild-type status and *TP53* mutant status were associated with poor survival (see Data Supplement Fig. 2) (Table 1). In multivariate analysis, only grade and CRS with HIPEC were independent predictors of survival while mutations in *TP53* (HR 1.38, 95%CI: 0.8–2.5, *P* = 0.278) and *GNAS* (HR 1.07, 95%CI: 0.5–2.2, *P* = 0.845) were not (Table 1 and Fig. 4). In an exploratory pairwise comparison between clinical (grade and CRS with HIPEC) and molecular factors (*GNAS* and *TP53* mutation status), only CRS + HIPEC with GNAS mutation status was found to be significant (Fig. 4).

**Table 1:** Univariate and multivariate analyses for overall survival (OS).

Variables	Category	Univariate analysis			Multivariate analysis^a^	
		OS (months) (95%CI)	HR (95%CI)	*P* value	HR (IC 95%)	*P* value
Age (years)	>60	47.0 (30.0, 63.9)	1.25 (0.7–2.1)	0.399		
	≤60	76.6 (58.1, 95.0)				
Grade^b^	1	137.8 (52.7, 223.0)	Ref.	Ref.	Ref.	
	2	75.8 (58.1, 93.4)	6.09 (2.2–16.7)	<0.001	5.7 (2.0–16.3)	0.001
	3	41.9 (27.3, 56.6)	11.6 (4.3–31.5)	<0.001	9.7 (3.3–28.8)	<0.001
CRS + HIPEC	Yes	120.0 (59.5–180.6)	0.35 (0.2–0.6)	<0.001	0.44 (0.3–0.7)	0.002
	No	40.3 (28.5–52.2)				
Chemotherapy	Yes	75.8 (55.6, 96.0)	1.47 (0.8–2.8)	0.242		
	No	Not reached				
RAS	Mutant	75.8 (44.8, 106.7)	0.94 (0.6–1.5)	0.792		
	Wild-type	76.2 (50.0, 102.4)				
GNAS	Mutant	Not reached	0.53 (0.3–0.9)	0.026	1.07 (0.5–2.2)	0.845
	Wild-type	61.0 (35.0, 87.1)				
TP53	Mutant	31.8 (16.4, 47.2)	2.06 (1.3–3.3)	0.002	1.38 (0.8–2.5)	0.278
	Wild-type	76.9 (59.4, 94.3)				
SMAD4	Mutant	74.0 (15.0, 133.0)	1.30 (0.8–2.3)	0.284		
	Wild-type	76.6 (42.3, 110.8)				

*CI* confidence interval, *OS* overall survival, Ref. reference.

^a^Multivariate analysis performed using factors significant in univariate analysis.

^b^Grades 1, 2 and 3 imply well, moderately and poorly differentiated tumour, respectively.

### Molecular comparison between AA and CRC

**Fig. 5:**
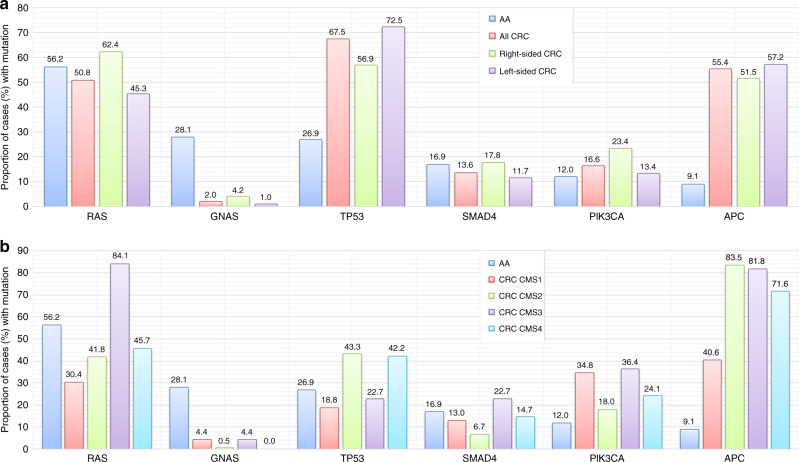
Mutations in Appendiceal Adenocarcinoma vs. Colorectal Cancer. Comparison (frequency %) of mutations among appendix cancer (AA) and colorectal cancer (CCR) w.r.t. sidedness (**a**) and consensus molecular subtypes (CMS) (**b**).

In comparative analyses with CRC, there were statistically significant differences among the major genes, such as *RAS*, *GNAS*, *TP53*, *PIK3CA* and *APC* between AA and right- and left-sided CRC (Fig. 5 and see Data Supplement Table S5). Notably, AAs had a significantly higher rate of GNAS (28.1% vs. 2%) and lower rate of TP53 (26.9% vs. 67.5%) and APC (9.1% vs. 55.4%). Similarly, there were statistically significant difference in mutation rates in relation to CMS subtypes (Fig. 5 and see Data Supplement Table S6). None of the CRC CMS groups appeared to have mutation profile similar to AAs. Appendiceal adenocarcinoma differed from right side, left side and CMS subtypes 1–4 in three or more of the six most frequently mutated genes.

## Discussion

The pivotal challenge of treating rare tumours stems from the absence of dedicated clinical and translational research. Appendiceal adenocarcinoma has been plagued with similar challenges. In the absence of large collaborative efforts, AAs have often been mixed with and treated as CRCs. Conceivably, this is the reason that over the past years, although survival for CRC has improved, survival in AA has shown no significant improvement.^30^ In the era of targeted therapies, this problem is amplified and the need for a clinico-molecular distinction is a critical unmet need.

Being the largest integrated clinico-molecular study of AA, these data confirm and augment the findings of earlier smaller cohorts.^7,8^ We conclude that AA is a distinct clinico-molecular entity from CRC and show that with regards to key genomic mutations, it does not resemble either right-sided CRC or any of the four CMS within CRC (see Data Supplement Tables S1, S5, S6). The strength of this study cohort lies in its substantial clinicopathological annotation, especially with regards to grade and tumour homogeneity. Prior cohorts have limited information on grade and have been a mix of AA, goblet cell carcinoids, pseudomyxoma peritonei, mucinous adenocarcinomas and signet-ring cell carcinomas. Due to the significant controversy regarding classification of epithelial appendix neoplasms, the consensus from Peritoneal Surface Oncology Group International (PSOGI) has highlighted the use of a uniform nomenclature and grade (well-, moderately- and poorly differentiated) characterisation for invasive AA.^31^ Somatic mutation profiles highlighted key molecular differences most notably much less frequent mutation in *APC* (9% vs. 55%) and *TP53* (27% vs. 67%), and much more frequent mutation in *GNAS* (28% vs. 2%) consistent with prior reports.^7–21^ The prevalence of microsatellite instability or deficient MMR in AAs is also remarkably low (0.9%) compared to right-sided CRC, where the incidence is about 7%. These results indicate a fundamental difference in disease biology between AAs and CRCs.^23^ Future efforts are needed to perform in-depth transcriptomic and translational profiling to study molecular subsets as has been done for CRC and to define biology-based subgroups that can be exploited therapeutically.^22,32^

Another advantage of the large cohort size is the ability to segregate AA by grade for subgroup analysis. Of note, given the critical role of determining histologic grade in the treatment planning of AA, whenever possible pathology should be reviewed by an experience pathologist (as was done in this case) given frequent over-interpretion by community pathologists.^33^ Consistent with the results of this study, low-grade AA have previously been shown to have a distinct molecular profile from high-grade tumours characterised by frequent mutation in *GNAS* and *KRAS* and absence of *TP53* mutation; given these molecular data and its distinct natural history characterised by indolent disease course, there is now overwhelming evidence to support the hypothesis that low-grade AA is a distinct disease entity from high-grade AA.^7,13,16,32^ This distinction has important clinical implications, given that low-grade AA tend to be unresponsive to traditional cytotoxic chemotherapy and in light of a recent large retrospective analysis (*N* = 639) showing no association of chemotherapy with improved OS.^34^ Additionally, new guidelines from the American Society of Colon & Rectal Surgeons now suggest avoiding chemotherapy for low-grade AA.^35^

Treatment modalities for AAs include CRS and HIPEC for surgically resectable disease and systemic therapy for unresectable cases.^36–39^ Systemic therapy used for AA patients in our cohort was similar to that for CRC patients, with most getting treated with 5-FU combinations with either oxaliplatin (FOLFOX) or irinotecan (FOLFIRI) with the addition of either an anti-EGFR, or anti-VEGF biological agent, which reflects current NCCN guidelines in CRC.^6^ Our population was intensively treated, with almost half of the patients submitted to HIPEC and second-line systemic therapy, possibly explaining the 5-year OS rates reported in the literature (46.0−58.5%).^2,30,40–43^

Leveraging the size and completeness of clinical annotation of this cohort allowed for multivariate analysis for the most important, independent predictors of survival. Only histologic grade (moderate differentiation: HR 5.7, *P* = 0.001; poor differentiation: HR 9.7, *P* < 0.001) and undergoing CRS with HIPEC (HR 0.44, *P* = 0.002) were independent predictors of survival. Complete CRS with HIPEC is critical in this disease with low-grade tumours while its role is still evolving in CRC as evident from the findings of PRODIGE 7 trial that showed no additional benefit to HIPEC after CRS.^44^ In our cohort, nearly all cases of CRS were combined with HIPEC; so it is not possible to determine the relative contribution of each as assessed by the PRODIGE 7 trial that showed no benefit from the addition of HIPEC to CRS in CRC. Mutations in *TP53* and *GNAS* were associated with worse and improved survival, respectively, when looked at individually. However, when adjusted for grade (low-grade tumours were highly enriched for *GNAS* mutation and high-grade tumours enriched for *TP53* mutation), no mutation was independently associated with survival, indicating that histologic grade is a more critical prognostic factor than the mutation status of any individual gene. Interestingly, even when restricting to just moderately differentiated tumours, *GNAS* and *TP53* were not predictive of survival. This suggests that AA is a network-based disease, arising not from a single dominant mutation but rather from the dysregulation of many genes converging to sustain an oncogenic transcriptional state. However, since we restricted our survival analysis to patients with advanced disease to ensure homogeneity, these results may not necessarily inform prognosis for early stage appendix tumours.

For both low-grade and high-grade tumours, opportunities for repurposing currently FDA-approved targeted therapies are limited given infrequent mutation in the common driver genes such as *BRAF*, *EGFR*, *HER2* and *KIT*. *KRAS*, once thought to be undruggable, has now been effectively targeted with covalent binders specifically to the cysteine in the G12C mutation. One potential target in low-grade tumours is the G-protein-coupled receptor GNAS. The *GNAS* R201C mutation has been shown to induce tumours when expressed with *KRAS* in mouse models suggesting it is a driver gene.^45^ Although *GNAS*-specific chemical inhibitors do not yet exist, the cystine in the R201C mutation could be targeted similarly to that in *KRAS* G12C. There were not any patients in this study treated with immune checkpoint inhibitors; however, given poor response rate of these agents in tumours with low mutation burden, as is the case for AA, these data do not present a strong case for clinical testing of immunotherapy in unselected AA patients without further pre-clinical data.

An additional important finding is the low sensitivity of blood-based NGS (ctDNA) particularly in low-grade AA. The majority of ctDNA tests for low-grade tumour showed no mutations, in stark contrast to the prevalence of mutated genes in tissue-based sequencing. These data are consistent with the data presented in prior reports of AA patients with ctDNA testing.^46^ In particular, while the absence of clinical annotation of grade in the Shaib et al. study limits its conclusions, the rate of mutations in key genes was far lower than our tissue-based cohort (*KRAS*: 18% vs. 56%; *GNAS*: 4% vs. 28%), indicating that in many cases mutations present in tumour failed detection using ctDNA. Interestingly, rate of *TP53* mutations was not lower (39% vs. 27%), which could potentially be influenced by clonal haematopoiesis or due to enrichment of their cohort with poorly differentiated tumours (grade was not known for these tumours). However, the low rate of mutations in blood-based analyses could be the result of poor shedding of tumour DNA in AAs, plausibly a grade-dependent phenomenon. Additionally, this may reflect differences between the liquid and tissue cohorts, for example disease burden, effect of prior surgery or chemotherapy, which can affect ctDNA dynamics. Contrary to what the authors concluded regarding the feasibility of ctDNA testing in AA, our findings show that ctDNA should be cautiously used in AA. However, due to the limited size of liquid biopsy cohort in our study, further studies with paired liquid and tumour biopsies are needed to validate this interesting observation prior to routine clinical use of liquid biopsies in AAs.

In conclusion, our data demonstrate that AA and CRC are distinct clinico-molecular entities and argues for dedicated research efforts in AAs. Grade outperforms key somatic mutations in predicting prognosis in this disease. Given the different molecular profiles, natural history and response to therapy, high-grade and low-grade AAs can themselves be considered unique disease entities.

## Supplementary information

Supplement

## Data Availability

All data in this study in a de-identified format are available upon request.
