# Supplementary material for: Integrated clinico-molecular profiling of appendiceal adenocarcinoma reveals a unique grade-driven entity distinct from colorectal cancer
Source: Br J Cancer. 2020 Jul 31;123(8):1262–70. doi: 10.1038/s41416-020-1015-3 (PMC7553941; doi:10.1038/s41416-020-1015-3)
Supplement: Supplementary file 1 — Supplement [file 41416_2020_1015_MOESM1_ESM.docx]

Supplementary Data

(Available at *BJC* online)

Integrated clinicomolecular profiling of appendiceal adenocarcinoma reveals a unique grade-driven entity distinct from colorectal cancer

Table of Contents

[**Table S1.** Comparison of appendix adenocarcinoma (AA) and colorectal cancer (CRC) 2](#_Toc44311599)

[**Table S2.** Baseline characteristics of patients with appendix adenocarcinoma in study. 0](#_Toc44311600)

[**Table S3.** Frequency of gene mutations in tumor tissue of appendix adenocarcinoma patients. 1](#_Toc44311601)

[**Figure S1.** Frequency (%) of mutations (> 3% incidence) in tumor tissue from patients with appendix cancer adenocarcinoma in the MDA-APP and MSK-IMACT cohorts. 0](#_Toc44311602)

[**Table S4:** Distribution of genes with respect to frequency (%) of mutations by grade (for genes with > 3% incidence) in patients with appendix adenocarcinoma. 1](#_Toc44311603)

[**Figure S2.** Kaplan-Meier overall survival (OS) curves for all patients (**Panel A**), and comparison between key factors, tumor grade (**Panel B**), cytoreductive surgery and HIPEC (**Panel C**), RAS mutation status (**Panel D**), GNAS mutation status (**Panel E**) and TP53 mutation status (**Panel F**). 2](#_Toc44311604)

[**Table S5.** Comparison (frequency %) of mutations among appendix adenocarcinoma (AA) and colorectal cancer (CRC) with respect to sidedness^1^. 3](#_Toc44311605)

[**Table S6.** Comparison (frequency %) of mutations among appendix adenocarcinoma (AA) and colorectal cancer (CRC) with respect to consensus molecular subtypes (CMS)**^1^**. 4](#_Toc44311606)

# **Table S1.** Comparison of appendix adenocarcinoma (AA) and colorectal cancer (CRC)

| Factor | Appendiceal Adenocarcinoma  (AA) | Colorectal Cancer  (CRC) | References (PMID) |
| --- | --- | --- | --- |
| Incidence |  |  | 26270447  31912902 |
| Age adjusted, US population | 1 per 100,000 | 40 per 100,000 |  |
| Patterns of Metastases |  |  | 12065772  27416752 |
| Peritoneal | 90% | 20% |  |
| Liver | 10% | 85% |  |
| Lung | 10% | 35% |  |
| Mucinous adenocarcinoma in metastatic setting |  |  | 26506400  22226571  22476818 |
| Prevalence | 60% | 10% |  |
| Prognostic Value (compared to non-mucinous) | Better prognosis | Unclear (? Poorer) |  |
| Signet-ring cell carcinoma in metastatic setting |  |  | 26506400  22476818 |
| Prevalence | 13% | 2% |  |
| Prognostic Value (compared to non-signet) | Poorer prognosis | Poorer prognosis |  |
| Treatment characteristics |  |  | 16389186  JCO-2018(LBA3503) |
| Benefit from heated intraperitoneal chemotherapy | Yes | Unclear |  |
| Genetic Differences |  |  | 23648460  20142816  30692096 |
| MSI-high | 2% | 15% |  |
| GNAS mutations | 30% | 2% |  |
| APC mutations | 10% | 75% |  |
| TP53 mutations | 40% | 70% |  |

# **Table S2.** Baseline characteristics of patients with appendix adenocarcinoma in study.

Restricted molecular testing performed due to limited tissue

N = 7

| Variable^1^ | N (266) | % |
| --- | --- | --- |
| Age (median) (years) | 53 (17 – 74) |  |
| Gender |  |  |
| Female | 144 | 54 |
| Male | 122 | 46 |
| TNM Stage |  |  |
| IV-A | 51 | 19 |
| IV-B | 123 | 47 |
| IV-C | 16 | 6 |
| IV-Unspecified | 73 | 28 |
| Tumor Grade |  |  |
| Well-differentiated | 54 | 28 |
| Moderately-differentiated | 55 | 29 |
| Poorly-differentiated | 83 | 43 |
| Sample for Sequencing |  |  |
| Tumor Tissue | 230 | 87 |
| Blood (ctDNA) | 24 | 9 |
| Both | 12 | 4 |
| Cytoreductive Surgery + HIPEC |  |  |
| Yes | 112 | 58 |
| No | 81 | 42 |
| Peritoneal Carcinomatosis Index (PCI) (median) (range) | 17 (2 – 39) |  |
| 1-10 | 19 | 21 |
| 11-20 | 33 | 36 |
| >20 | 40 | 43 |
| Cytoreduction Score (CCS) |  |  |
| 0 | 60 | 55 |
| 1 | 26 | 24 |
| 2 | 20 | 19 |
| 3 | 2 | 2 |
| Systemic Chemotherapy |  |  |
| Yes | 142 | 74 |
| No | 51 | 26 |
| Median lines of therapy | 2 |  |
| Regimen used in first-line^2^ |  |  |
| FOLFOX/CAPOX + Bevacizumab | 58 | 41 |
| FOLFOX/CAPOX | 51 | 36 |
| FOLFIRI + Bevacizumab | 9 | 7 |
| Others | 24 | 17 |

**Abbreviations:** HIPEC, heated intraperitoneal chemotherapy; N, number

1. Some variables have missing values and percentages reflect patients with available data.
2. Others include: FOLFIRI, Cape/5FU + Bev, Cape/5FU, Bev, FOLFOXIRI + Bev

# **Table S3.** Frequency of gene mutations in tumor tissue of appendix adenocarcinoma patients.

| Gene | MDA  N | MDA  % | MSK  N | MSK  % | All  N | All  % |
| --- | --- | --- | --- | --- | --- | --- |
| KRAS | 87 | 51.5 | 49 | 67.1 | 136 | 56.2 |
| GNAS | 43 | 25.4 | 25 | 34.2 | 68 | 28.1 |
| TP53 | 38 | 22.5 | 27 | 37.0 | 65 | 26.9 |
| SMAD4 | 30 | 17.8 | 11 | 15.1 | 41 | 16.9 |
| PIK3CA | 22 | 13.0 | 7 | 9.6 | 29 | 12.0 |
| APC | 14 | 8.3 | 8 | 11.0 | 22 | 9.1 |
| ATM | 13 | 7.7 | 4 | 5.5 | 17 | 7.0 |
| KIT | 15 | 8.9 | 0 | 0.0 | 15 | 6.2 |
| FBXW7 | 4 | 2.4 | 5 | 6.8 | 9 | 3.7 |
| MET | 9 | 5.3 | 0 | 0.0 | 9 | 3.7 |
| BRAF | 7 | 4.1 | 1 | 1.4 | 8 | 3.3 |
| SMAD2 | 1 | 0.6 | 5 | 6.8 | 6 | 2.5 |
| AKT1 | 2 | 1.2 | 3 | 4.1 | 5 | 2.1 |
| CDH1 | 5 | 3.0 | 0 | 0.0 | 5 | 2.1 |
| NRAS | 5 | 3.0 | 0 | 0.0 | 5 | 2.1 |
| CDKN2A | 4 | 2.4 | 0 | 0.0 | 4 | 1.7 |
| ARID2 | 3 | 1.8 | 0 | 0.0 | 3 | 1.2 |
| CTNNB1 | 3 | 1.8 | 0 | 0.0 | 3 | 1.2 |
| MYC | 3 | 1.8 | 0 | 0.0 | 3 | 1.2 |
| STK11 | 3 | 1.8 | 0 | 0.0 | 3 | 1.2 |
| Gene list for gene mutation seen in 2 patients (0.8%): AR, AXL, BLM, BRCA1, BRCA2, CEBPA, FLT1, FLT3, FOXP1, GLI1, IDH1, JAK3, KDR, MLH1, MLL2, MPL, RB1, RUNX1T1, SLX4, TSC2. | | | | | | |
| Gene list for gene mutation seen in 1 patient (0.4%): ABL1, ARID1A, ARID1B, ATR, BAP1, BCORL1, C11orf30, CBL, CCND1, CCND2, CCND3, CCNE1, CDKN2B, CIITA, CREBBP, CSF1R, CTNNA1, DICER1, EGFR, ERBB2, ESR1, EXT2, FANCA, FANCC, FANCD2, FGFR3, GRIN2A, HGF, HRAS, IRF2, IRF4, IRS2, KDM5A, KDM5C, KDM6A, MAPK1, MAP3K1, MED12, MEF2B, MLL, MSH6, MTOR, MUTYH, MYD88, NF1, NTRK1, PALB2, PDGFRA, PDGFRB, PIK3R1, PIK3R2, POLD1, POLE, PRKDC, RAD21, RET, RPS6KB1, RPTOR, SGK1, SMO, SPTA1, TAF1, TET2, ZNF217. | | | | | | |

**Abbreviations:** MDA, MDACC-APP cohort; MSK, MSK-IMPACT cohort

# **Figure S1.** Frequency (%) of mutations (> 3% incidence) in tumor tissue from patients with appendix cancer adenocarcinoma in the MDA-APP and MSK-IMACT cohorts.


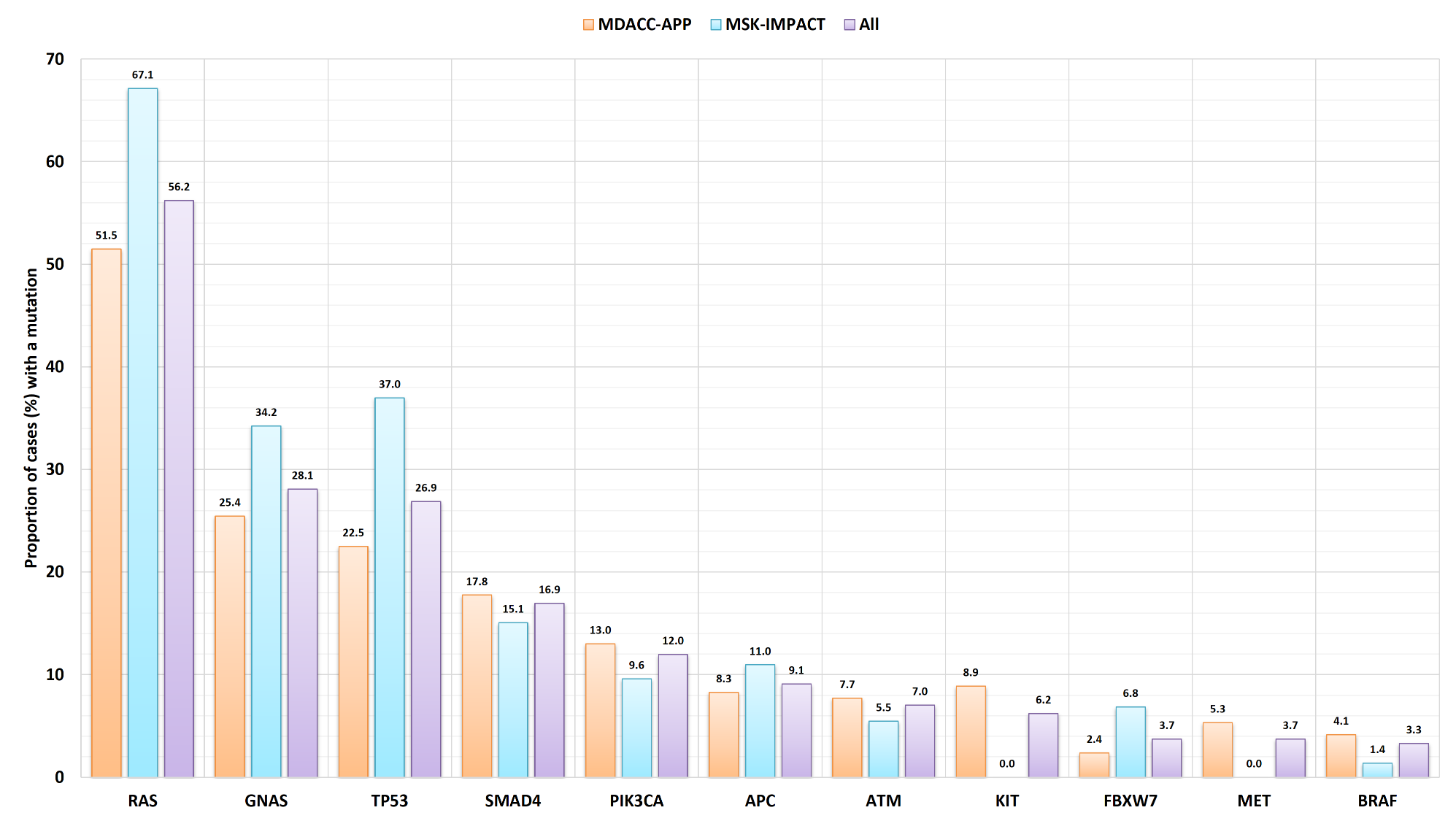


# **Table S4:** Distribution of genes with respect to frequency (%) of mutations by grade (for genes with > 3% incidence) in patients with appendix adenocarcinoma.

| Grade | Well  (N = 39) | | Mod.  (N = 50) | | Poor  (N = 79) | | Well v Mod. | | | Well v Poor | | | Mod. V Poor | | | All |
| --- | --- | --- | --- | --- | --- | --- | --- | --- | --- | --- | --- | --- | --- | --- | --- | --- |
| Gene | **MUT (N)** | **%** | **MUT (N)** | **%** | **MUT (N)** | **%** | **OR** | **95%CI** | **P^1^** | **OR** | **95%CI** | **P^1^** | **OR** | **95%CI** | **P^1^** | **P^2^** |
| RAS | 27 | 69.2 | 32 | 64.0 | 32 | 40.5 | 1.3 | 0.5-3.0 | 0.6563 | 3.3 | 1.4-7.2 | 0.0058 | 2.6 | 1.2-5.4 | 0.0116 | 0.0033 |
| GNAS | 19 | 48.7 | 16 | 32.0 | 8 | 10.1 | 2.0 | 0.8-4.7 | 0.13 | 7.7 | 2.9-20.0 | <0.0001 | 4.2 | 1.6-10.6 | 0.0026 | <0.0001 |
| TP53 | 3 | 7.7 | 13 | 26.0 | 22 | 27.8 | 0.2 | 0.1-0.9 | 0.0290 | 0.2 | 0.1-0.7 | 0.0154 | 0.9 | 0.4-2.0 | 0.84 | 0.0383 |
| SMAD4 | 5 | 12.8 | 10 | 20.0 | 14 | 17.7 | 0.6 | 0.2-1.9 | 0.41 | 0.7 | 0.2-2.0 | 0.60 | 1.2 | 0.5-2.8 | 0.82 | 0.59 |
| PIK3CA | 5 | 12.8 | 2 | 4.0 | 15 | 19.0 | 2.4 | 0.6-9.3 | 0.29 | 0.6 | 0.2-1.8 | 0.45 | 0.2 | 0.0-0.7 | 0.0157 | 0.18 |
| APC | 0 | 0.0 | 8 | 16.0 | 6 | 7.6 | 0.1 | 0.0-0.9 | 0.0398 | 0.3 | 0.0-2.1 | 0.42 | 2.3 | 0.7-7.4 | 0.15 | 0.35 |
| ATM | 4 | 10.3 | 6 | 12.0 | 3 | 3.8 | **Note:** Analyses limited to six most frequently mutated genes due to low numbers in other genes limits power to detect any meaningful difference. | | | | | | | | | |
| KIT | 5 | 12.8 | 4 | 8.0 | 6 | 7.6 |  |  |  |  |  |  |  |  |  |  |
| FBXW7 | 0 | 0.0 | 3 | 6.0 | 1 | 1.3 |  |  |  |  |  |  |  |  |  |  |
| MET | 0 | 0.0 | 2 | 4.0 | 7 | 8.9 |  |  |  |  |  |  |  |  |  |  |
| BRAF | 1 | 2.6 | 2 | 4.0 | 4 | 5.1 |  |  |  |  |  |  |  |  |  |  |

**Abbreviations:** Mod., moderately; MUT, mutant; OR, odds ratio; CI, confidence interval

1. Fisher’s exact test
2. Chi-square test

# **Figure S2.** Kaplan-Meier overall survival (OS) curves for all patients (**Panel A**), and comparison between key factors, tumor grade (**Panel B**), cytoreductive surgery and HIPEC (**Panel C**), RAS mutation status (**Panel D**), GNAS mutation status (**Panel E**) and TP53 mutation status (**Panel F**).

**
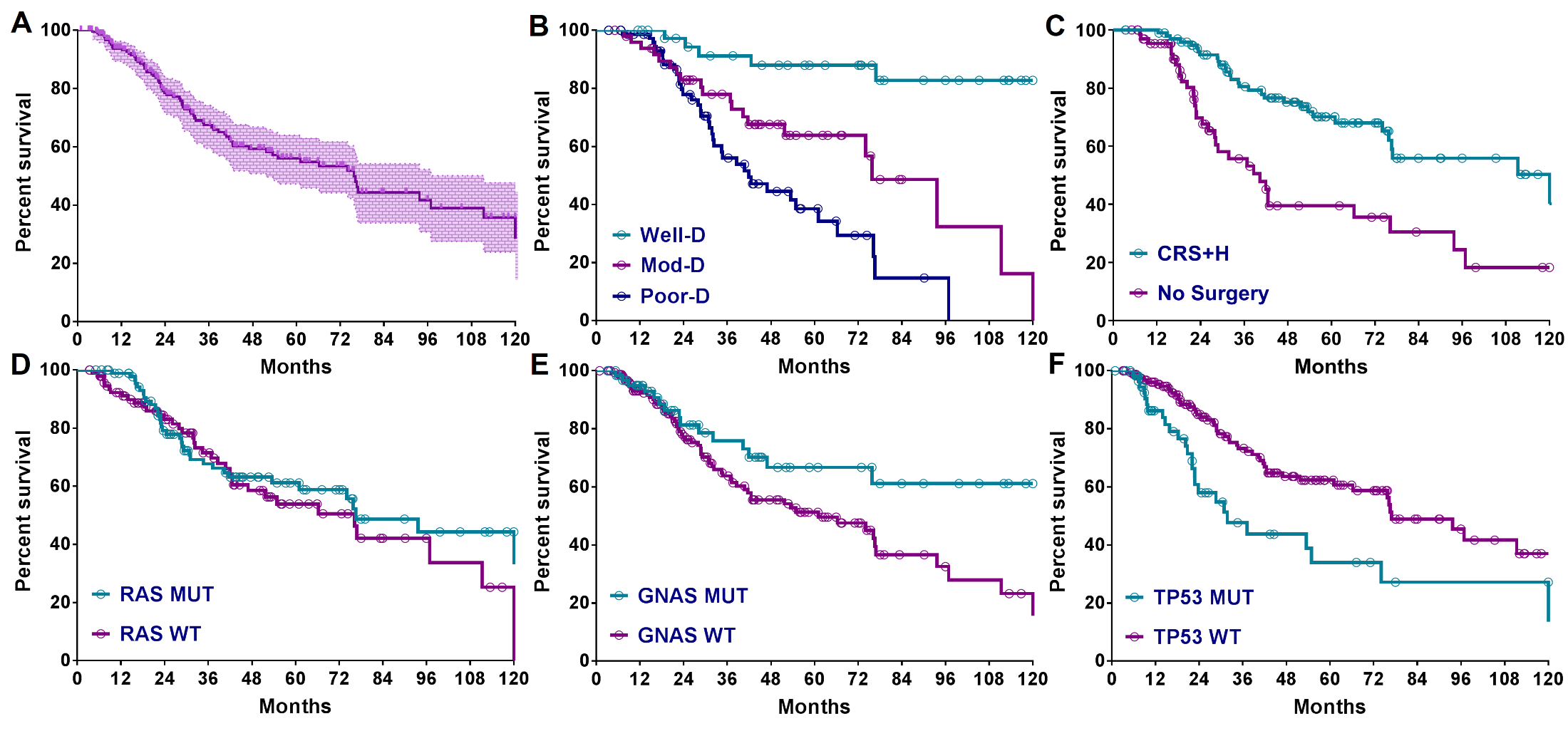
**

**Abbreviations:** CRS+H, cytoreductive surgery and HIPEC; D, differentiated; Mod, moderately; MUT, mutant; Poor, poorly; WT, wild type

# **Table S5.** Comparison (frequency %) of mutations among appendix adenocarcinoma (AA) and colorectal cancer (CRC) with respect to sidedness^1^.

| Gene | AA | All  CRC | OR  (95%CI) | P | Right  Side | OR  (95%CI) | P | Left  Side | OR  (95%CI) | P |
| --- | --- | --- | --- | --- | --- | --- | --- | --- | --- | --- |
| RAS | 56.2 | 50.8 | 1.2  (1.0,1.6) | 0.1083 | 62.4 | 0.8  (0.6,1.0) | 0.0882 | 45.3 | 1.5  (1.2,2.0) | 0.0016 |
| GNAS | 28.1 | 2.0 | 18.8  (12.9,27.8) | <0.0001 | 4.2 | 9.0  (5.8,13.9) | <0.0001 | 1.0 | 37.6  (22.6,64.0) | <0.0001 |
| TP53 | 26.9 | 67.5 | 0.2  (0.1,0.2) | <0.0001 | 56.9 | 0.3  (0.2,0.4) | <0.0001 | 72.5 | 0.1  (0.1,0.2) | <0.0001 |
| SMAD4 | 16.9 | 13.6 | 1.3  (0.9,1.8) | 0.1747 | 17.8 | 0.9  (0.6,1.3) | 0.7769 | 11.7 | 1.5  (1.1,2.2) | 0.022 |
| PIK3CA | 12.0 | 16.6 | 0.7  (0.4,1.0) | 0.0689 | 23.4 | 0.4  (0.3,0.7) | <0.0001 | 13.4 | 0.9  (0.6,1.3) | 0.6151 |
| APC | 9.1 | 55.4 | 0.1  (0.0,0.1) | <0.0001 | 51.5 | 0.1  (0.0,0.1) | <0.0001 | 57.2 | 0.1  (0.0,0.1) | <0.0001 |

**Abbreviations:** OR, odds ratio; CI, confidence interval

1. Top 6 most commonly mutated genes shown. Green boxes indicate significant *P* value.

# **Table S6.** Comparison (frequency %) of mutations among appendix adenocarcinoma (AA) and colorectal cancer (CRC) with respect to consensus molecular subtypes (CMS)**^1^**.

| Gene | AA | CMS1 | OR (95%CI) | P | CMS2 | OR (95%CI) | P | CMS3 | OR (95%CI) | P | CMS4 | OR (95%CI) | P |
| --- | --- | --- | --- | --- | --- | --- | --- | --- | --- | --- | --- | --- | --- |
| RAS | 56.2 | 30.4 | 2.9  (1.7,5.1) | 0.0002 | 41.8 | 1.8  (1.2,2.6) | 0.0029 | 84.1 | 0.2  (0.1,0.6) | 0.0004 | 45.7 | 1.5  (1.0,2.4) | 0.0706 |
| GNAS | 28.1 | 4.4 | 8.6  (2.7,26.8) | <0.0001 | 0.5 | 75.0  (13.7,762.3) | <0.0001 | 4.4 | 8.6  (2.2,36.8) | 0.0003 | 0.0 | NE  (12.2,NE) | <0.0001 |
| TP53 | 26.9 | 18.8 | 1.6  (0.8,3.1) | 0.2088 | 43.3 | 0.5  (0.3,0.7) | 0.0004 | 22.7 | 1.2  (0.6,2.5) | 0.7098 | 42.2 | 0.5  (0.3,0.8) | 0.0051 |
| SMAD4 | 16.9 | 13.0 | 1.4  (0.6,3.0) | 0.5773 | 6.7 | 2.8  (1.5,5.5) | 0.0012 | 22.7 | 0.7  (0.3,1.5) | 0.3919 | 14.7 | 1.2  (0.7,2.2) | 0.6474 |
| PIK3CA | 12.0 | 34.8 | 0.3  (0.1,0.5) | <0.0001 | 18.0 | 0.6  (0.4,1.0) | 0.0786 | 36.4 | 0.2  (0.1,0.5) | 0.0002 | 24.1 | 0.4  (0.2,0.8) | 0.0051 |
| APC | 9.1 | 40.6 | 0.1  (0.1,0.3) | <0.0001 | 83.5 | 0.0  (0.0,0.0) | <0.0001 | 81.8 | 0.0  (0.0,0.1) | <0.0001 | 71.6 | 0.0  (0.0,0.1) | <0.0001 |

**Abbreviations:** OR, odds ratio; CI, confidence interval

1. Top 6 most commonly mutated genes shown. Green boxes indicate significant P value.
